# Menahydroquinone-4 Prodrug: A Promising Candidate Anti-Hepatocellular Carcinoma Agent

**DOI:** 10.3390/diseases3030150

**Published:** 2015-07-22

**Authors:** Munechika Enjoji, Daisuke Watase, Kazuhisa Matsunaga, Mariko Kusuda, Nami Nagata-Akaho, Yoshiharu Karube, Jiro Takata

**Affiliations:** Faculty of Pharmaceutical Sciences, Fukuoka University, 8-19-1 Nanakuma, Jonan-ku, Fukuoka 814-180, Japan; E-Mails: watase@adm.fukuoka-u.ac.jp (D.W.); k-matsu@fukuoka-u.ac.jp (K.M.); kusuda@adm.fukuoka-u.ac.jp (M.K.); akaho@adm.fukuoka-u.ac.jp (N.N.-A.); karube@adm.fukuoka-u.ac.jp (Y.K.); jtakata@fukuoka-u.ac.jp (J.T.)

**Keywords:** vitamin K, hepatocellular carcinoma, anti-cancer drug, drug delivery, menaquinone-4

## Abstract

Recently, new therapeutics have been developed for hepatocellular carcinoma (HCC). However, the overall survival rate of HCC patients is still unsatisfactory; one of the reasons for this is the high frequency of recurrence after radical treatment. Consequently, to improve prognosis, it will be important to develop a novel anti-tumor agent that is especially effective against HCC recurrence. For clinical application, long-term safety, together with high anti-tumor efficacy, is desirable. Recent studies have proposed menahydroquinone-4 1,4-bis-*N,N*-dimethylglycinate hydrochloride (MKH-DMG), a prodrug of menahydroquinone-4 (MKH), as a promising candidate for HCC treatment including the inhibition of recurrence; MKH-DMG has been shown to achieve good selective accumulation of MKH in tumor cells, resulting in satisfactory inhibition of cell proliferation in des-γ-carboxyl prothrombin (DCP)-positive and DCP-negative HCC cell lines. In a spleen-liver metastasis mouse model, MKH-DMG has been demonstrated to have anti-proliferation and anti-metastatic effects *in vivo*. The characteristics of MKH-DMG as a novel anti-HCC agent are presented in this review article.

## 1. Introduction

Intracellular levels of vitamin K and its homologs are significantly lower in most hepatocellular carcinomas (HCCs) as compared with background non-tumor areas [1]; vitamin K-dependent carboxylation reactions are impaired in HCC cells. Vitamin K can inhibit the growth of HCC cells in a dose-dependent manner. However, vitamin K uptake is lower in HCC cells relative to normal hepatocytes *in vitro* [2]. These findings support the hypothesis that differences in the ability to absorb vitamin K lead to the individual sensitivity of HCC to vitamin K. Nowadays, the vitamin K_2_ (menaquinone) homolog menaquinone-4 (MK-4) is used for the treatment of osteoporosis, and its long-term safety has been confirmed [3,4,5,6]. There is a possibility that MK-4 can suppress the progression of HCC [7,8,9,10,11,12]. In some small-scale studies, MK-4 treatment has been reported to reduce the onset of HCC in patients with liver cirrhosis and HCC recurrence after curative surgical resection or radiofrequency ablation [13,14]. Indeed, MK-4 is expected to inhibit *de novo* carcinogenesis, HCC proliferation and HCC recurrence with long-term safety. However, no significant inhibiting effect was proven in a large-scale, double-blind and randomized control study [15]. A meta-analysis of randomized controlled trials, failed to confirm significantly better tumor recurrence-free survival at one year, and there was no beneficial effect on the overall survival [16].

Because the anti-HCC effect of MK-4 may be dependent on the delivery of its metabolite, menahydroquinone-4 (MKH), it is hypothesized that effective delivery of MKH to HCC cells leads to inhibition of HCC proliferation, metastasis and recurrence. However, MKH itself has easily oxidizable characteristics and is unsuitable for clinical use. In our previous studies, menahydroquinone-4 1,4-bis-*N,N*-dimethylglycinate hydrochloride (MKH-DMG), the ester derivative of MKH, showed excellent MKH delivery potential regarding the liver without the reductive activation process of MK-4 to MKH [17,18]. To improve the prognosis of patients with HCC, an agent with high anti-tumor effectiveness and a good safety profile needs to be developed. Recent studies suggest that MKH-DMG has promising anti-HCC characteristics, such as intracellular MKH delivery and inhibition of tumor progression [19].

## 2. MKH Delivery System in HCC Cells

**Figure 1:**
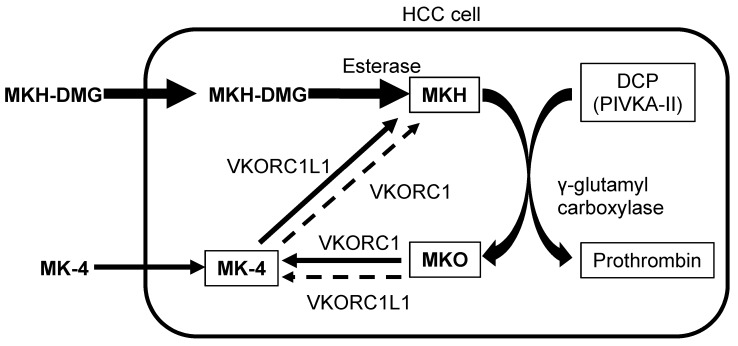
Schema of MKH delivery system in HCC cells. MKH-DMG, menahydroquinone-4 1,4-bis-*N,N*-dimethylglycinate hydrochloride; MK-4, menaquinone-4; MKH, menahydroquinone-4; MKO, menaquinone-4 epoxide; DCP, des-γ-carboxyl prothrombin; PIVKA-II, protein induced by vitamin K absence-II; VKORC1, vitamin K 2,3-epoxide reductase complex subunit 1; VKORC1L1, VKORC1 like-1; HCC, hepatocellular carcinoma.

The schema for the conversion process associated with the MKH delivery system in HCC cells is shown in Figure 1. Des-γ-carboxy prothrombin (DCP) also known as the protein induced by vitamin K absence-II (PIVKA-II), an abnormal prothrombin with incomplete carboxylation, is a HCC-specific tumor marker [20,21]. Additionally, DCP functions as a predictive factor for vascular invasion, metastasis and tumor progression; it is associated with the poor prognosis of HCC patients [22,23,24,25,26,27]. The vitamin K content in HCC cells has the ability to restrict DCP production [1,28,29,30]. MKH, a fully reduced form of MK-4, is a cofactor of γ-glutamyl carboxylase (GGCX) that converts glutamate residue into the γ-carboxyglutamate residue of vitamin K-dependent proteins such as prothrombin [31,32,33]. Therefore, ancillary to vitamin K-dependent carboxylation, MKH is stoichiometrically converted into menaquinone-4 epoxide (MKO). Utilization of MKH is restricted in HCC tissue. Because MKH availability regulates the rate of carboxylation [34], reduction of MKH availability in HCC cells may result in the increase in DCP.

MKH-DMG, an MKH prodrug, is hydrolyzed into MKH by esterase after its uptake by cells. GGCX is required for its activity and depends upon MKH, which is generated mainly by vitamin K 2,3-epoxide reductase complex, subunit 1-like 1 (VKORC1L1). VKORC1L1 promotes the reduction of MK-4 to MKH and supports vitamin K hydroquinone-mediated intracellular antioxidation, which is critical for cell survival [35]. In our previous studies, MKH-DMG effectively supplied MKH to HCC cells without reductive activation [17,18].

## 3. Inhibition of HCC Cell Proliferation by MKH-DMG

MKH-DMG has an inhibitory effect regarding cell proliferation in HCC cell lines *in vitro*. In our cell viability assay, MKH-DMG suppressed the proliferation of HCC cells in a time- and dose-dependent manner both in DCP-positive (PLC/PRF/5 and Hep3B) and DCP-negative (SK-Hep1) cell lines [19]. A rapid and intensive suppression effect was evident at 48 h after MKH-DMG administration at a concentration of 20 μM (Table 1). In contrast, the effect of MK-4 was clearly weaker and the suppression of cell proliferation was not distinct until 72 h at a concentration of ≥40 μM (Table 1). MKH-DMG showed significantly lower IC_50_ values (14–37 μM) and exhibited 4–18 times stronger proliferation-suppressing activity than MK-4. When cell injury was estimated by LDH release into the medium, no indication of injury was evident during MKH-DMG treatment. MK-4 is usually prescribed at a dose of 45 mg/day (three times per day) with a good long-term safety profile in the treatment of osteoporosis patients. At this dose, the maximum plasma concentration is 1 μM [36]; in a previous distribution study of oral MK-4 administration, the hepatic concentration was >10 times higher than the plasma concentration [37]. These results indicate that the IC_50_ values of MKH-DMG used in the treatment of HCC cell lines may be clinically reasonable levels, and that MKH-DMG is potentially a promising anti-HCC agent available for safe clinical use.

**Table 1:** Effects for HCC cells (Reference [19]).

Treatment	Inhibition of Cell Proliferation (*in vitro*)	MKH Delivery (*in vitro*)	Downregulation of DCP (*in vitro*)	HCC inhibition (*in vivo*) *
MKH-DMG (concentration)	obvious (20 μmol/L)	obvious (25 μmol/L)	obvious (10 μmol/L)	obvious (0.2 μmol/day)
MK-4 (concentration)	weak (40 μmol/L)	weak (25 μmol/L)	obvious (10 μmol/L)	-

* MKH-DMG was dissolved in drinking water (40 μmol/L) and provided *ad libitum*.

## 4. Effective MKH Delivery into HCC Cells by MKH-DMG

Inhibition of HCC proliferation, metastasis and recurrence by MKH-DMG and MK-4 are determined by their ability to deliver MKH to HCC cells. It is difficult to measure MKH levels accurately in HCC cells because of its highly oxidative characteristics regarding MK-4. However, MKO levels can be substituted for MKH levels (Figure 1). MKH delivery via MKH-DMG or MK-4 (both at a concentration of 25 μM) to HCC cell lines has been estimated by means of intracellular MKO and MK-4 levels [19]. MKH-DMG treatment induced a rapid and time-dependent increase in intracellular MKO and MK-4 levels (Table 1). In this case, MK-4 is an oxidation product of MKH (Figure 1). MKH-DMG is taken up by HCC cells and effectively converted into MKH. Conversely, MK-4 administration did not lead to an increase in MKO and MK-4 levels (Table 1), which supports the evidence that the MK-4 uptake rate was lower in HCC cells relative to normal hepatocytes [2]. When the AUC of the intracellular concentration *versus* time profile was determined, the AUC_0-72 h_ values for MKH were 3.5–15 times higher after MKH-DMG treatment than those after MK-4 treatment, regardless of whether DCP-positive or DCP-negative cell lines were used. These results indicate that MKH-DMG administration is an effective method for the delivery of MKH to HCC cells.

MKH-DMG as an MKH prodrug shows satisfactory cell-membrane permeability and is effectively hydrolyzed into MKH by esterase present in HCC cells, leading to the rapid and intensive inhibition for HCC proliferation. Perhaps the uptake process for MKH-DMG is different to that for MK-4. Investigation of MKH-DMG uptake including transporter system may be an important target for future research.

## 5. Downregulation of DCP by MKH-DMG

In a recent investigation, DCP/PIVKA-II was reported to act as a growth and metastasis factor for HCC, and to exacerbate its prognosis [27]. Therefore, depression of DCP may be a prospective target for developing a novel treatment against DCP-positive HCC. This therapeutic strategy is applicable to MKH-DMG administration, which can achieve sufficiently high MKH delivery. The effect of MKH-DMG regarding the DCP level in a DCP-positive PLC/PRF/5 cell line was evaluated by measuring the DCP concentration in culture media at 72 h after treatment [19]. DCP levels had clearly decreased after MKH-DMG (10 μM) and MK-4 (10 μM) treatments (2.0 ± 0.0 mAU/mL, and 1.3 ± 0.6 mAU/mL, respectively) relative to untreated controls (43 ± 3.6 mAU/mL). However, as described above, cell proliferation was only suppressed after MKH-DMG treatment and not after MK-4 treatment. Because growth inhibition caused by MKH-DMG has also been demonstrated in DCP-negative HCC cells, DCP suppression may not be important for MKH-DMG and MKH regarding the inhibition of HCC growth. Other vitamin K-dependent proteins, with the exception of DCP, may be critical regarding the effect of MKH-DMG, and such vitamin K-dependent proteins should be explored.

## 6. Induction of Cell-Cycle Arrest by MKH-DMG

The underlying mechanism concerning the effect of MKH-DMG on HCC cell growth suppression should be investigated. Generally, cell-cycle arrest has been considered to be primarily associated with the anti-proliferative action of MK-4 [7,8,9,11,37,38]. Aberrant expression of NF-κB is linked to cyclin D1 [10], and to the onset and progression of HCC tumorigenesis [38]. Because both cyclin D1 and NF-κB are associated with cellular migration [39,40], downregulation of NF-κB and cyclin D1 by MKH-DMG treatment may contribute to the inhibition of HCC cell growth and invasion.

In our analysis, it is suggested that G_1_/S arrest is one of the mechanisms involved in the inhibition of HCC cell proliferation induced by MKH-DMG [19]. Flow cytometric analysis of MKH-DMG-treated PLC/PRF/5 cells *in vitro* revealed an increase in G_1_ phase cells and a decrease in S phase cells. In Western blot analysis, the expression of cell cycle-related proteins (cyclin D1, cyclin D3 and CDK4) decreased and was completely suppressed after 48 h of MKH-DMG treatment in both DCP-positive (PLC/PRF/5 and Hep3B) and DCP-negative (SK-Hep-1) HCC cell lines. Conversely, similar treatment using MK-4 induced only a slight decrease in the levels of these proteins. Additionally, NF-κB was downregulated by MKH-DMG treatment in these HCC cell lines, which might have resulted from effective MKH delivery to the tumor cells using MKH-DMG.

## 7. Anti-Proliferation Effect of MKH-DMG *in Vivo*

The clinical benefit of MKH-DMG treatment has been demonstrated using xenografted human HCC cells *in vivo* [19]. In a preceding pharmacokinetic study after oral administration of MKH-DMG, MKH-DMG was found to be delivered to and absorbed by hepatocytes in the same esterized form, and then converted to MKH *in vivo*. Using this MKH delivery system, the effects of MKH-DMG in combating hepatic metastasis and the proliferation of HCC cells (PLC/PRF/5) were examined in a mouse spleen-liver metastasis model (nu/nu mice). Macroscopic findings of liver tumors were estimated at 50 days after transplantation of PLC/PRF/5 cells in MKH-DMG treated and untreated groups. Increases in both liver weight and the percentage of cancer surface area were significantly suppressed in the MKH-DMG treatment group as compared with the untreated group. In the MKH-DMG treatment group, plasma DCP, which is produced by HCC cells, was not detected, although liver metastasis could not be suppressed completely. These findings mean that MKH is definitely delivered to the metastasized HCC cells and that DCP is suppressed by MKH; they suggest the possibility that MKH-DMG could function as an anti-HCC agent in humans. However, subsequent investigation is required to show whether or not a similar effect can be demonstrated after MKH-DMG treatment of human HCC.

## 8. Conclusions

The effects of MKH-DMG and MK-4 regarding HCC cells are summarized in Table 1. Reduced uptake of therapeutic agents by cancer cells is one of the important factors affecting resistance to chemotherapy. Consequently, the development of compounds with tumor-specific cell penetration properties is still a primary focus of cancer research. Effective supply of MKH, an active form of MK-4, is a promising approach concerning the suppression of HCC growth with low toxicity. MKH-DMG, a MKH prodrug in esterized form, can deliver substantial amounts of MKH to HCC cells, and can suppress HCC proliferation effectively both *in vitro* and *in vivo*. The anti-HCC effect of MKH-DMG may be the result of cell-cycle arrest with downregulation of NF-κB and cyclin D1 expression. MKH-DMG is a promising new candidate for suppression of the onset of HCC, its recurrence and metastasis without significant adverse effects.
